# Serological Outcome in the First Months of Life of Children Born to Mothers with SARS-CoV-2 Infection during Pregnancy

**DOI:** 10.3390/children11091095

**Published:** 2024-09-06

**Authors:** Gemma Pons-Tomàs, Irene Martínez-de-Albeniz, María Ríos-Barnés, Anna Gamell, Sílvia Simó-Nebot, Sol Balsells-Mejía, María Hernández-García, Maria Melé-Casas, Emilia Sánchez, Manuel Monsonis, Amadeu Gené, Marta López, Dolors Salvia, Juan-José Garcia-García, Claudia Fortuny, Victoria Fumadó

**Affiliations:** 1 Pediatric Department, Hospital Sant Joan de Déu, University of Barcelona, 08950 Barcelona, Spain; gemma.pons@sjd.es (G.P.-T.); maria.hernandezg@sjd.es (M.H.-G.); maria.mele@sjd.es (M.M.-C.); or juanjo_garcia@ub.edu (J.-J.G.-G.); 2 Infectious Diseases and Microbiome, Institut de Recerca Sant Joan de Déu (IRSJD), 08950 Barcelona, Spain; maria.rios@sjd.es (M.R.-B.); annamaria.gamell@sjd.es (A.G.); silvia.simo@sjd.es (S.S.-N.); or cfortuny@ub.edu (C.F.); 3 Infectious and Imported Diseases Department, Hospital Sant Joan de Déu, 08950 Barcelona, Spain; irene.malbeniz@gmail.com; 4 Consorcio de Investigación Biomédica en Red de Epidemiología y Salud Pública (CIBERESP), 28029 Madrid, Spain; 5 Research Promotion and Management Department, Hospital Sant Joan de Déu, 08950 Barcelona, Spain; sol.balsells@sjd.es; 6 Blanquerna School of Health Sciences, Universitat Ramon Llull, 08022 Barcelona, Spain; emiliasr@blanquerna.url.edu; 7 Department of Microbiology, Hospital Sant Joan de Déu, 08950 Barcelona, Spain; manuel.monsonisc@sjd.es (M.M.); amadeu.gene@sjd.es (A.G.); 8 Neonatology Service, Hospital Clinic La Maternitat—BCNatal Research, 08028 Barcelona, Spain; lopezro@clinic.cat (M.L.); dsalvia@clinic.cat (D.S.); 9 Department of Surgery and Medical-Surgical Specialties, Faculty of Medicine and Health Sciences, University of Barcelona, 08036 Barcelona, Spain

**Keywords:** SARS-CoV-2, children, pregnancy, antibodies transplacental transmission, serology persistence, transmission

## Abstract

Background: The objective of this study is to analyze the transplacental transmission of SARS-CoV-2 antibodies, their persistence in newborns, the factors that may influence this transmission, and the protection these antibodies confer over time. Methods: This prospective cohort was conducted in a tertiary pediatric hospital in the Barcelona Metropolitan Region, Spain. It included neonates born to mothers who had SARS-CoV-2 infection during pregnancy or delivery between August 2020 and January 2022. We followed the recruited children for at least six months, and blood tests were performed to determine the presence of SARS-CoV-2 antibodies. Results: A total of 101 children were recruited. Among the serologies performed on children under three months of age, 44/82 were positive (53.7%). Newborns whose mothers presented more severe disease exhibited higher seropositivity odds (coefficient 9.747; *p* = 0.002). There were increased preterm deliveries when maternal infection occurred closer to the time of delivery. No severe SARS-CoV-2 infections were detected in children during the follow-up. Conclusions: Slightly more than half of the SARS-CoV-2 serologies performed in the first three months were positive. This appears to confer protection during early childhood. The severity of maternal infection is the most significant factor influencing the transmission of antibodies in children born to unvaccinated mothers.

## 1. Introduction

The Coronavirus Disease 2019 (COVID-19) pandemic was declared in March 2020. Since then, millions of pregnant women have been infected by SARS-CoV-2, raising concerns about the severity of the infection in pregnant women as well as its impact on the fetus and, subsequently, on the newborn.

A large part of the pediatric population with SARS-CoV-2 infection presents as asymptomatic or with mild disease [1]. However, during the neonatal period, the infection seems to be more severe [2,3] compared to children and adolescents. Although infection in the neonatal period is rare, some symptomatic neonates require admission to the Neonatal Intensive Care Unit (NICU) [4]. Thus, it is essential to ascertain whether and how often the infection is transmitted from pregnant women to their offspring. Additionally, it is crucial to determine whether SARS-CoV-2 antibodies are transmitted from mother to child during pregnancy, the duration of positivity, and their effect on protection against the disease. Although studies have described the impact of SARS-CoV-2 infection during pregnancy on the child until the first months of life [5], these children’s long-term clinical and serological evolution has yet to be studied.

In other infections, it has been observed that acute infection alters transplacental antibody transfer by an unknown mechanism. The transmission of specific antibodies for SARS-CoV-2 appears lower than other respiratory infections, such as influenza or whooping cough [6]. It has been proposed that changes in the placenta caused by SARS-CoV-2 infection may explain this decrease in transmission [7]. However, despite this reduction in specific antibody transfer, a median transplacental antibody transfer efficiency of 81% was reported, measured in the cord blood of the newborns born to infected mothers during pregnancy [7].

The clinical progression of COVID-19 infection has been described as more severe in symptomatic pregnant women compared to non-pregnant women of the same age [8]. In addition, the infection has been associated with an increased risk of pregnancy complications, such as preeclampsia or premature birth [2,3,9,10]. Some authors suggest that this may be partly due to placental damage [11]. This increased risk of complications among pregnant women underscores the importance of understanding the progression of SARS-CoV-2 infection in this population. In Spain, SARS-CoV-2 vaccination of pregnant women began in May 2021.

The primary objectives of this study are to determine the seroprevalence of SARS-CoV-2 antibodies over time (2–4 weeks and 6 months of life) among newborns and infants born to mothers with confirmed infection during pregnancy and to describe the factors associated with seropositivity in this population.

The secondary objectives are to determine whether there is an association between the time of infection and severity of the mother’s infection, time of delivery (term or preterm), and birth weight, to compare seroprevalence rates among children born to mothers who received at least one dose of SARS-CoV-2 vaccine with children born to unvaccinated mothers, and to conduct clinical follow-up to detect possible infections or other complications during the first months of life.

## 2. Materials and Methods

### 2.1. Study Description

This prospective cohort study included neonates born to mothers with confirmed SARS-CoV-2 infection during pregnancy (confirmed by positive nasopharyngeal PCR or antigen test) or delivery (confirmed by positive nasopharyngeal PCR).

We offered participation to neonates born in two tertiary hospitals (Hospital Clínic La Maternitat and Hospital Sant Joan de Déu) in the Barcelona Metropolitan Region (Spain). During the study period, Hospital Sant Joan de Déu attended 5082 deliveries, and Hospital Clínic La Maternitat, 4669 deliveries.

Participants were recruited from August 2020 to January 2022, and the enrolled children were followed up in infectious diseases outpatient clinics of Hospital Sant Joan de Déu, where clinical and serological monitoring was performed.

Concerning clinical follow-up, the participants and their families attended the infectious diseases outpatient clinics when the children were 1, 3, and 6 months old, although visit timings were occasionally adjusted based on family availability. Pediatricians conducted both an anamnesis and a physical examination during these visits. At the first visit, we collected information from medical records regarding maternal history, SARS-CoV-2 vaccination status, the timing and severity of maternal SARS-CoV-2 infection, pregnancy and delivery details, and neonatal anthropometry at birth. If the medical records did not provide all the necessary information, we obtained it through anamnesis with the caregivers. Additionally, the feeding method (breastfeeding or formula feeding) was determined through anamnesis.

For serological monitoring, we performed blood tests to determine the presence and persistence of SARS-CoV-2 antibodies. The first test was conducted during the first month of life, and a second serology was performed at around six months of age. Those with persistent positive serology were retested at around nine months of age. Some parents consented to the use of data but not to perform venipuncture. These children were also included in the study.

Therefore, clinical (with on-site visits) and serological follow-up of the offspring were performed until they were at least six months old.

The vaccination of pregnant women against SARS-CoV-2 started during the recruitment period; antibody transmission was analyzed separately based on whether the mother had received any vaccine doses during pregnancy.

### 2.2. Hospital Protocols and Definitions

According to hospital protocol, PCR testing was performed on all pregnant women at delivery. For those who tested positive, a nasopharyngeal PCR test was performed on the newborn 2 h after birth, with a follow-up PCR required 24–48 h later to rule out infection. In cases where the mother tested positive for SARS-CoV-2, the newborn was kept at least two meters away from the mother and, whenever possible, placed in an incubator while awaiting the newborn’s PCR results. Droplet and contact isolation precautions were implemented. The mother and accompanying caregivers had to use surgical masks and practice rigorous hand hygiene. Breastfeeding was recommended whenever clinically feasible and per the mother’s preferences.

Severe maternal infection was determined by one or more of the following criteria: severe pneumonia with failure of one or more organs, oxygen saturation (SaO_2_) < 90%, a respiratory rate ≥ 30 breaths per minute, or the need for vasopressors. It also included cases of respiratory distress, sepsis, or septic shock. Moderate infection was characterized by mild pneumonia that did not require oxygen therapy. Mild infection included localized symptoms in the upper respiratory tract and non-specific symptoms, such as fever or muscle pain. Hospital admission was indicated in cases of moderate and severe infection.

### 2.3. Laboratory

Serum samples obtained by venipunctures were tested by a chemiluminescent immunoassay of microparticles (CMIA) (Abbott^®^). SARS-CoV-2-specific IgM against the spike glycoprotein and IgG against the nucleocapsid were initially determined using the Abbott SARS-CoV-2 IgG assay on the ARCHITECT i Systems following the manufacturer’s instructions. From January 2021 onwards, IgG against spike glycoprotein was measured using the SARS-CoV-2 IgG II Quant assay. Anti-nucleocapsid IgG (until January 2021) was considered positive with an index >1.4 (qualitative result). For anti-spike IgG (from January 2021), antibody binding units per milliliter (AU/mL) were quantified, considering a positive result defined as >50 AU/mL.

### 2.4. Statistics

A descriptive analysis of the database was conducted initially. Continuous variables were described using means and standard deviations for normally distributed data or medians and interquartile ranges (IQR) for non-normally distributed data. Categorical variables were described using frequency tables. To assess the association between the risk factors and seropositivity rates, logistic regression was used with mixed models to account for clustering (some patients had more than one antibody determination). Age at serology was considered an a priori confounder, and standardized age was included in the analysis to control for its effect. Ordinal independent variables were transformed into numerical values to be included in the models. Linear regression models explored the association between antibody titer on the logarithmic scale and risk factors. Normality and homoscedasticity of the residuals were checked, and robust methods were used to estimate the coefficients in cases where necessary. Statistical significance was set at *p* < 0.05. Statistical analyses were performed using version 4.3.1 of R in the RStudio program (version 2022.02.0+443).

### 2.5. Ethics

This study was conducted in accordance with the Declaration of Helsinki, and the protocol was approved by the Ethics Committee of Hospital Sant Joan de Déu (EOM-09-21). Signed informed consent was obtained for all the participants, from one or both parents, or their legal guardians.

## 3. Results

During the study period, 101 children from 99 mothers were recruited (2 women gave birth to twins).

### 3.1. Maternal History and Acute SARS-CoV-2 Infection

Among the enrolled children, 56 (55.4%) were boys. The median age of the mothers was 34 years (IQR 30–37), and 14/99 mothers (14.14%) had a relevant pre-existing medical condition, with metabolic syndrome being the most common (4/99, 4.04%) (Table 1).

Overall, nine women received at least one dose of the SARS-CoV-2 vaccine during pregnancy. SARS-CoV-2 infection was diagnosed in 12 women (12.12%) during the first trimester of gestation, 23 women (23.23%) during the second trimester, and 42 women (42.42%) during the third trimester. At delivery, SARS-CoV-2 RNA was detected in 20 women (20.2%). In two cases, the timing of infection was unknown. Most women (83/99, 83.8%) had asymptomatic or mild infection. Overall, 11 mothers required hospital admission, 2 of whom were admitted to the ICU). Eight women required respiratory support: six with nasal cannula oxygen therapy, one with non-invasive respiratory support, and one with invasive respiratory support. None of the women required hemodynamic support. The descriptions and analyses of the severity of infection, moment of delivery, and birth weight concerning the moment of infection are summarized in Table 2 and Table 3. The severity of symptoms was lower the closer the infection was to delivery (*p* = 0.007) (Table 3). Excluding women with infection at the time of delivery, no association was found between the timing of infection and the severity of symptoms.

### 3.2. Pregnancy, Delivery, and Complications at Birth and during Follow-Up

Among the total deliveries, 8 out of 99 (8.1%) were pre-term. The odds of preterm delivery increased significantly as the time of the mother’s infection approached delivery (*p* = 0.021) (Table 3). Regarding birth weight, 12/101 (11.9%) neonates were small for gestational age (SGA), 12/101 (11.9%) were large for gestational age (LGA), and 77/101 (76.2%) were adequate for gestational age (AGA). Complications were observed in 21/99 (21.2%) pregnancies and 6/101 (5.9%) births. The detailed complications and other pregnancy and neonatal characteristics are summarized in Table 1.

At the hospital where the study was conducted, according to the protocol in force at the time, neonates were not separated from mothers with SARS-CoV-2 infection at the time of delivery (except in two cases where the mother required respiratory support in the ICU). Nasopharyngeal PCR was performed in 15 of the 20 newborns born to mothers with COVID-19 infection at delivery (as medically indicated to rule out infection in the newborn). Two of these newborns tested positive for SARS-CoV-2: one of them was asymptomatic, while the other required admission to PICU during the neonatal period due to severe hyperbilirubinemia. Additionally, IgM was detected in three patients in the first blood test (one of these had a positive PCR, and the other two had negative PCR results). None of the included children exhibited COVID-19 symptoms at birth, and none required hospital admission due to SARS-CoV-2 infection during the follow-up period.

During follow-up, almost all the children (96/101, 95%) were breastfed; 58/101 (57.4%) were exclusively breastfed, and 37/101 (37%) were mixed breastfed and formula-fed.

### 3.3. Seroprevalence in Newborns Born to Mothers with SARS-CoV-2 Infection during Pregnancy or Delivery

**Figure 1:**
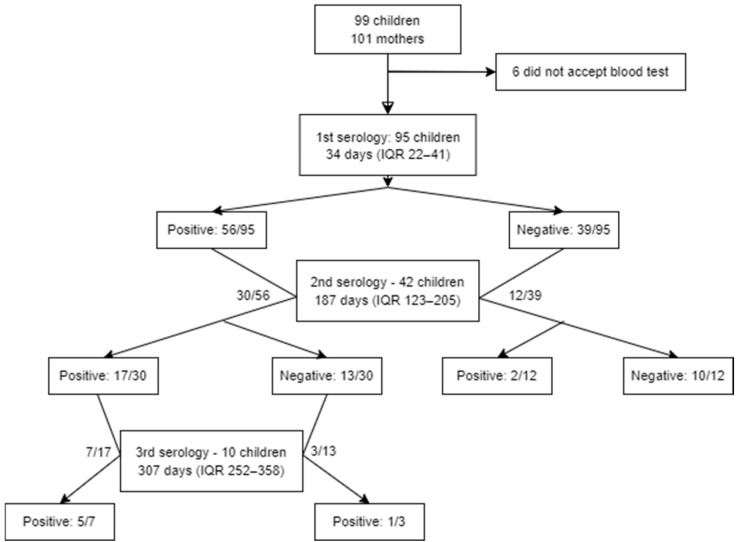
Flow diagram of study participants. During follow-up, some participants did not attend the visit, while others did not authorize venipuncture. Out of protocol, in 3 participants, a 3rd serology was performed after a first positive and a second negative serology.

A total of 147 serologies were performed on 95 patients during the follow-up. In six cases, the family did not consent to venipuncture. A flow diagram of the study participants is presented in Figure 1.

#### 3.3.1. Antibody Transmission from Unvaccinated Mothers

To investigate transplacental antibody transmission from mother to newborn and its persistence over time, nine children whose mothers had received a dose of the vaccine during pregnancy, four children who tested positive for PCR or IgM at birth, and three children who presented a positive serology following an initial negative result were excluded. After these exclusions, 118 serological tests from 80 patients were analyzed.

Table 4 presents the associations between the presence of anti-SARS-CoV-2 IgG antibodies and factors that may have influenced transplacental antibody transmission, such as age at serology, the timing of maternal infection, and the severity of the maternal symptoms.

**Figure 2:**
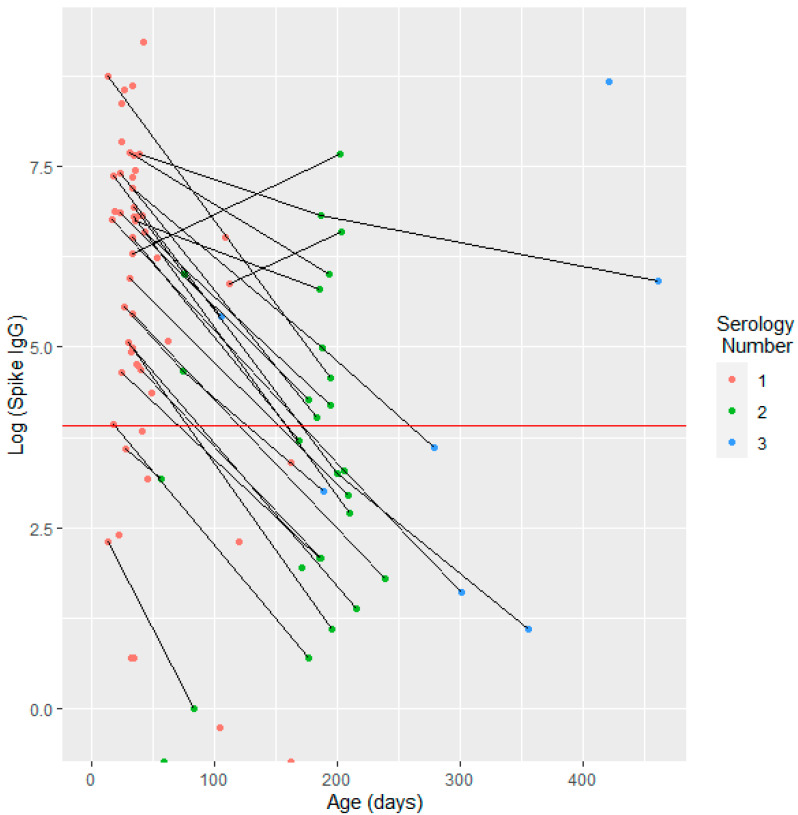
Antibody titer on a logarithmic scale as a function of age at venipuncture for children born to unvaccinated mothers.

After categorizing the serological results into three age groups (0 to 3 months, 4 to 6 months, and over six months), it was observed that 44/82 (53.66%) serological tests were positive in the 0 to 3 months age group. There was a significant decline in antibody positivity with increasing patient age (coefficient: −6.451; *p* < 0.001) (Table 4). Similarly, Figure 2 illustrates the relationship between age and IgG levels, showing a decrease in IgG levels as the patient’s age increases.

Figure 2 depicts the anti-Spike IgG titer concerning the age of the children, with a total of 94 serological tests presented. The anti-Spike IgG values are displayed on a logarithmic scale. Each line connects data points for the same patient, illustrating changes in the anti-Spike IgG levels at different ages. The red line indicates the cutoff value for positive serology, defined as an anti-Spike IgG level of 50 AU/mL or higher.

Regarding the timing of infection, we observed a higher presence of antibodies when the infection occurred closer to delivery. However, this trend did not hold when the infection was diagnosed at the time of delivery, corresponding to the lowest antibody transmission level. Although higher antibody transmission was observed when infection occurred during the second or third trimester, no significant differences were found when analyzed without considering the delivery timing (Table 4).

Additionally, increased odds of positive IgG were observed in children whose mothers experienced a severe infection (coefficient = 9.747; *p* = 0.002).

The anti-spike IgG antibody titer was analyzed in 94 serological tests (with the remainder either not determined or measured for anti-nucleocapsid IgG). There was strong evidence of an association between age at the time of serology and antibody titer (*p* < 0.001). No differences in antibody titer were found in relation to the timing or severity of maternal infection (Table 5).

#### 3.3.2. Seropositivity from Vaccinated Mothers

A total of 9/99 (9.10%) women received at least one dose of SARS-CoV-2 vaccine during pregnancy. We analyzed 15 serological samples from 10 infants born to these vaccinated mothers. Compared to the children of unvaccinated mothers, those born to vaccinated mothers had higher odds of IgG positivity (coefficient = 19.465; *p* < 0.001) and higher median antibody titers (coefficient = 1075.828; *p* < 0.001) (Table 4 and Table 5).

### 3.4. Clinical Follow-Up

During the study period, a total of 515 medical visits were conducted. Follow-up lasted a median of 5.1 months (IQR: 1–8 months), with a median of 2.8 clinic visits per patient (IQR 2–4). At birth and within the first three months of life, four patients with probable acute infection were identified: one with a positive PCR result, one with positive PCR and IgM, and two with positive IgM. During the subsequent follow-up, two additional children were found to have positive SARS-CoV-2 PCR results (at 6 and 8 months) during the evaluations for upper respiratory tract infections with mild symptoms. No patients in the cohort experienced severe SARS-CoV-2 infection.

## 4. Discussion

Our study found that 53.66% of anti-SARS-CoV-2 IgG serologies performed in children under three months of age born to mothers with a history of COVID-19 during pregnancy or delivery were positive. No SARS-CoV-2 infections were observed during the first six months of life.

Previous studies have suggested that the efficiency of transplacental transmission of antibodies against SARS-CoV-2 is lower compared to other viruses [6]. However, adequate antibody transmission was observed in 87% (95% CI, 78–93%) of the newborns in the cohort studied by Flannery D. et al., with IgG detected in cord blood [12]. In contrast, Corsi et al. found slightly lower antibody transmission, with 50.5% of SARS-CoV-2 positive IgG in newborns [13], which is consistent with our findings.

It has been described that transplacental antibody transmission is reduced when maternal infection occurs at the time of delivery, whereas higher transmission rates are observed when infection occurs during the second trimester [14,15,16,17]. Although we observed the lowest antibody transmission when infection occurred at delivery, no significant differences were detected.

Another factor that may affect transmission is the severity of maternal infection. Some studies have shown that pregnant women with moderate or severe infections have higher plasma IgG concentrations compared to women with asymptomatic or mild disease [12,18]. Considering that a good correlation has been observed between the antibody level of the pregnant woman and the antibody level in cord blood and the neonate [14,18], a higher transfer of antibodies to the neonate can be expected in cases where the mother has had a moderate or severe infection. Our study also found increased transplacental IgG transfer in women with symptomatic infection compared to those without symptoms (*p* = 0.002). However, we did not find significant differences in antibody titers in children born to these two groups.

Since the initiation of COVID-19 vaccination in pregnant women, it has been observed that maternal IgG levels against SARS-CoV-2 are higher in vaccinated women compared to unvaccinated women with a history of SARS-CoV-2 infection [19]. In our study, although only nine women were vaccinated and vaccination had not yet been widely implemented at the time of recruitment, we observed a significantly higher proportion of positive serologies and higher antibody titers in children born to vaccinated mothers. However, these estimates are uncertain due to the small sample size of vaccinated women.

Regarding neonatal and maternal outcomes, SARS-CoV-2 infection in pregnant women may increase the risk of preeclampsia through mechanisms leading to vascular disease [20,21]. This, in turn, can be a significant cause of prematurity [22,23,24] and low birth weight, outcomes associated with the severity of maternal infection [23]. Due to the limited number of pregnant women with severe disease in our cohort, a detailed analysis of these outcomes is challenging. Nonetheless, we observed increased preterm birth when maternal infection occurred closer to delivery.

Despite extensive research, the risk of maternal–fetal transmission of SARS-CoV-2 remains unclear, partly due to the lack of standardized criteria for diagnosing vertical infection. Reports of minimal or no transmission of maternal acute infection have been documented [25,26,27,28]. A published meta-analysis found infection transmission in 1.8% of newborns (95% CI: 1.2–2.5%) (n = 14271, from 140 cohort studies) [29]. This is consistent with our data, where only two newborns had positive PCR results at birth. The remaining children showed no symptoms compatible with SARS-CoV-2 infection. Pregnant women are widely considered a high-risk population for SARS-CoV-2 infection. However, the severity of infection during pregnancy appears to impact the mother more than the neonate [5], unlike other infections, such as the Zika virus [30]. Flannery et al. studied 209 pregnant women who tested positive for perinatal SARS-CoV-2 (16 days before or 3 days after delivery); 6 of 191 newborns tested positive for SARS-CoV-2, but none presented symptoms attributable to the infection [5].

None of the infants in our cohort presented clinical manifestations consistent with SARS-CoV-2 infection at birth. The complications observed in neonates and reasons for NICU admission were not related to SARS-CoV-2 infection but rather to the severity of maternal infection, which is consistent with what other authors have already described [31].

As observed in other infections, the transplacental passage of antibodies following maternal infection seems to confer protection to the infant. Some studies have followed neonates born to mothers with a history of COVID-19 up to 1–2 months of age and found no SARS-CoV-2 infections, suggesting passive immunity [5,28]. One of the strengths of our study is the median follow-up period of 5 months, during which only two children were found to be infected with SARS-CoV-2, and both after 6 months of life.

Our study has several limitations. Firstly, the timing of the blood tests varied among the children, which complicates the analysis of antibody persistence of the predefined ages. Secondly, losses to follow-up represented a significant limitation, as conducting strict follow-up and invasive tests on healthy children proved challenging. Additionally, we cannot distinguish whether antibodies detected in children a few months after birth are due to the persistence of transplacental antibodies or to undetected SARS-CoV-2 infections.

## 5. Conclusions

In conclusion, slightly more than half of the patients born to mothers with a history of SARS-CoV-2 infection during pregnancy or at delivery had antibodies against SARS-CoV-2 at three months. No SARS-CoV-2 infections were detected among these children in the first six months of life. This suggests that such antibodies may offer protection to children in early childhood. The severity of maternal infection appears to be the most significant factor influencing the transmission of antibodies in children born to unvaccinated mothers.

## Figures and Tables

**Table 1:** Maternal medical history, pregnancy, and neonatal period complications.

Maternal Medical History (N = 99)	14/99 (14.14%)
Metabolic syndrome Diabetes mellitus (2), myocardial infarction (1), hypertension (1), obesity (1)	5 (5%)
Autoimmune disease Lupus, autoimmune cholangitis, ulcerative colitis, rheumatoid arthritis	4 (4%)
Hypothyroidism	3 (3%)
Asthma	1 (1%)
Mental disorder Depression, obsessive–compulsive disorder	2 (2%)
Uterine myoma	1 (1%)
Cervical conization (human papillomavirus infection)	1 (1%)
Pregnancy complications (N = 99)	21/99 (21.21%)
Preterm rupture of membranes	3 (3%)
Gestational diabetes	6 (5.9%)
Chorioamnionitis	2 (2%)
Gestational cholestasis	2 (2%)
Gestational anemia	1 (1%)
Severe hyperemesis gravidarum	1 (1%)
Preeclampsia	5 (4.9%)
Structural malformations	1 (1%)
Type of delivery (N = 101)	
Eutocic	62 (61.4%)
Vaginal dystocic	10 (9.9%)
Cesarean section	26 (25.7%)
Unknown	3 (3%)
Complications at birth (N = 101)	6 (5.9%)
Respiratory depression (needing respiratory resuscitation with non-invasive ventilation)	4
Pneumothorax	1
Fracture of the parietal bone	1
Admission to NICU (immediately after delivery) (N = 101)	7 (6.9%)
Prematurity	2
Hyperbilirubinemia (phototherapy)	2
Suspected infection	1
Fetal acidosis	1
Social cause (mother admitted to ICU for severe SARS-CoV-2 infection)	1
Hospital admission (after discharge from hospital but <28 days of age)	2 (2%)
Hyperbilirubinemia (phototherapy)	2

Values are expressed as absolute numbers and percentages in parentheses.

**Table 2:** Description of severity of infection, moment of delivery, and birth weight concerning the moment of infection.

	Moment of Maternal Infection				
	1st trimester n = 12 (12.1%)	2nd trimester n = 23 (23.2%)	3rd trimester n = 42 mothers (42.4%) (44 newborns)	Delivery n = 20 (20.2%)	Unknown n = 2 (2%)
Severity of maternal infection					
Asymptomatic (26/99)	2 (16.7%)	3 (13.0%)	7 (16.7%)	14 (70.0%)	0 (0%)
Mild infection (57/99)	8 (66.7%)	19 (82.6%)	24 (57.1%)	6 (30.0%)	0 (0%)
Moderate/severe pneumonia (10/99)	1 (8.3%)	1 (4.4%)	8 (19.1%)	0 (0%)	0 (0%)
Unknown (6/99)	1 (8.3%)	0 (0%)	3 (7.1%)	0 (0%)	2 (100%)
Moment of delivery					
Term (91/99; 91.9%)	12 (100%)	23 (100%)	40 (90.91%)	16 (80%)	0 (0%)
Pre-term (8/99; 8.1%)	0	0	4 (9.09%)	4 (20%)	0 (0%)
Birth weight					
SGA (12/101; 11.9%)	1 (8.33%)	2 (8.7%)	7 (15.91%)	2 (10%)	0 (0%)
LGA (12/101; 11.9%)	1 (8.33%)	2 (8.7%)	6 (13.64%)	2 (10%)	1 (50%)
AGA (77/101; 76.2%)	10 (83.33%)	19 (82.6%)	31 (70.45%)	16 (80%)	1 (50%)

Mild infection: upper respiratory infection; moderate/severe infection: moderate (not requiring oxygen) or severe (requiring respiratory support) pneumonia. LGA: large for gestational age; SGA: small for gestational age; AGA: appropriate for gestational age.

**Table 3:** Analysis of severity of infection, moment of delivery, and birth weight in relation to the time of infection.

	Severity of Maternal Infection Linear Regression			Time of Delivery Logistic Regression			Birth Weight: SGA (Compared to AGA) Logistic Mixed Model			Birth Weight: LGA (Compared to AGA) Logistic Mixed Model		
	Coef.	SE	*p*	Coef.	SE	*p*	Coef.	SE	*p*	Coef.	SE	*p*
Intercept	1.136	0.127	<0.001	−5.307	1.490	<0.001	−2.189	0.716	0.002	−3.685	3.575	0.303
Moment of maternal infection	−0.178	0.065	0.007	1.362	0.590	0.021	0.138	0.354	0.697	0.178	0.576	0.758

Coef.: coefficient; SE: standard error.

**Table 4:** Antibody positivity throughout the follow-up according to the time of serology, time and severity of maternal infection, and the mother’s vaccination status.

	IgG Negative (n = 62)	IgG Positive (n = 56)	Coefficient	Standard Error	*p*-Value
Age of serology determination					
Intercept			−0.960	1.795	0.593
0–3 months	38/82 (46.34%)	44/82 (53.66%)	−6.451	1.249	<0.001
3–6 months	9/13 (69.23%)	4/13 (30.77%)			
>6 months	15/23 (65.22%)	8/23 (34.78%)			
Moment of infection					
Intercept			13.296	3.353	<0.001
Age			−10.988	2.645	<0.001
1st trimester	8/11 (72.73%)	3/11 (27.27%)	−10.930	2.602	<0.001
2nd trimester	14/38 (36.84%)	24/38 (63.16%)			
3rd trimester	25/51 (49.02%)	26/51 (50.98%)			
Delivery	15/16 (93.75%)	1/16 (6.25%)			
Association between time of infection and IgG positivity, excluding time of delivery			0.429	2.335	0.854
Severity of infection					
Intercept			−8.704	3.156	<0.001
Age			−6.552	1.452	<0.001
Asymptomatic	25/32 (78.12%)	7/32 (21.88%)	9.747	3.114	0.002
Mild infection	28/68 (41.18%)	40/68 (58.82%)			
Moderate/severe pneumonia	5/12 (62.5%)	7/12 (58.3%)			
Vaccination status					
Intercept			−1.091	1.745	0.532
Age			−6.238	1.062	<0.001
Not vaccinated	62/118 (52.54%)	56/118 (47.46%)	19.465	4.906	<0.001
Vaccinated	1/15 (6.67%)	14/15 (93.33%)			

Mixed-effects logistic regression was used.

**Table 5:** Anti-spike IgG levels according to the timing of serology, the timing and severity of maternal infection, and the mother’s vaccination status.

	Anti-Spike IgG Levels (UI/mL)	Coefficient	Standard Error	*p*-Value
Age of serology determination				
Intercept		583.648	85.223	<0.001
0–3 months	398 (IQR 85.2–1256)	−1.647	0.572	0.007
3–6 months	30 (IQR 5.75–110)			
>6 months	61.5 (IQR 13.2–318)			
Moment of infection				
Intercept		483.655	142.098	0.001
Age		−1.670	0.578	0.006
1st trimester	112 (IQR 27.5–165)	71.402	75.549	0.349
2nd trimester	118 (IQR 24–568)			
3rd trimester	364 (IQR 27.8–916)			
Delivery	103 (IQR 8–390)			
Severity of infection				
Intercept		480.341	122.987	<0.001
Age		−1.723	0.582	0.005
Asymptomatic	30 (IQR 6–236)	132.497	94.716	0.168
Mild infection	160 (IQR 37–977)			
Moderate/severe pneumonia	355 (IQR 6.5–634.5)			
Vaccination status				
Intercept		826.424	116.801	<0.001
Age		−3.255	0.754	<0.001
Not vaccinated (94)	147 (IQR 24.5–862)	1075.828	221.386	<0.001
Vaccinated (15)	1245 (IQR 182–10044)			

Mixed-effects linear regression was used. Random effects associated with the mother and the patient were used.

## Data Availability

The datasets generated and analyzed during the current study are not publicly available because individual privacy could be compromised but are available from the corresponding author upon reasonable request.
